# Aspects épidémio-cliniques des cancers du sein au Service d´Oncologie de Fianarantsoa, Madagascar de 2011 à 2018

**DOI:** 10.11604/pamj.2021.38.264.20031

**Published:** 2021-03-15

**Authors:** Mampionona Ranaivomanana, Nomeharisoa Rodrigue Emile Hasiniatsy, Hajanirina Rakotomahenina, Florine Rafaramino

**Affiliations:** 1 Service d´Oncologie, Centre Hospitalier Universitaire de Tambohobe Fianarantsoa, Faculté de Médecine de Fianarantsoa, Fianarantsoa, Madagascar,; 2 Service d´Oncologie et Soins Palliatifs, Centre Hospitalier de Soavinandriana, Antananarivo, Madagascar,; 3 Service de Gynécologie et Obstétrique, Centre Hospitalier Universitaire de Tambohobe Fianarantsoa, Faculté de Médecine de Fianarantsoa, Fianarantsoa, Madagascar,; 4 Faculté de Médecine d´Antananarivo, Université d´Antananarivo, Antananarivo, Madagascar

**Keywords:** Cancer du sein, épidémiologie, examen clinique, Breast cancer, epidemiology, clinical examination

## Abstract

**Introduction:**

à notre connaissance, il s´agit d´une première étude épidémiologique des cancers du sein à Fianarantsoa. Notre objectif était de décrire les caractéristiques épidémio-cliniques de ces cancers au Service d´Oncologie de Fianarantsoa.

**Méthodes:**

il s´agissait d´une étude rétrospective descriptive au Service d´Oncologie du Centre Hospitalier Universitaire de Tambohobe pendant 8 ans (2011 à 2018). Nous avons inclus toutes les patientes atteintes de cancer du sein avec une confirmation cytologique et/ou histologique. Les paramètres étudiés étaient l´âge, la profession, les antécédents familiaux de cancer du sein, la ménarche, la ménopause, la parité, la prise de contraception orale, le tabagisme, les circonstances de découverte, les symptômes mammaires, les signes d´extension, la localisation tumorale et le stade de la maladie.

**Résultats:**

nous avons inclus 62 patientes d´âge moyen de 52,83 ± 10,47 ans. Les femmes au foyer constituaient 39% (n = 24) des cas. Aucune patiente n´avait une ménarche précoce. La ménopause tardive était survenue chez 6,45% (n = 4) et des antécédents familiaux de cancer du sein étaient retrouvés chez 8,06% (n = 5). La prise de tabac à chiquer a été retrouvée chez 17,74% (n = 11) des cas. Les symptômes mammaires étaient observés dans 95,2% (n = 59) des cas. Le quadrant supéro-externe était touché dans 53,23% (n = 33) des cas. Le stade III s´observait dans 55% (n = 34) des cas et le stade IV dans 32% (n = 20).

**Conclusion:**

diagnostiqués à un stade avancé, les facteurs de risque de cancer du sein étaient peu observés.

## Abstract (en)

**Introduction:**

we conducted the first epidemiological study of patients with breast cancers living in Fianarantsoa. The purpose of this study was to describe the epidemiology and clinical features of these patients in the Department of Oncology Fianarantsoa.

**Methods:**

we conducted a retrospective and descriptive study in the Department of Oncology at the University Hospital Center of Tambohobe over a period of 8 years (2011-2018). All patients with breast cancer diagnosed based on cytological and/or histological examination were included. The parameters studied were: age, occupation, a family history of breast cancer, menarche, menopause, parity, use of oral contraceptives, smoking, circumstances leading to detection, breast symptoms, signs of locoregional and distant spread, tumour site and stage of the disease.

**Results:**

the study included 62 patients with an average age of 52.83 ± 10.47 years. Housewives accounted for 39% (n = 24) of cases. No patient had an early menarche. Late menopause had occurred in 6.45% (n = 4) of patients and a family history of breast cancer was found in 8.06% (n = 5) of patients. Chewing tobacco was used by 17.74% (n = 11) of patients. Patients reporting breast symptoms accounted for 95.2% (n = 59). Breast cancers had affected the upper outer quadrant in 53.23% (n = 33) of cases. Stage III breast cancer was diagnosed in 55% (n = 34) of cases and stage IV in 32% (n = 20) of cases.

**Conclusion:**

in patients diagnosed with cancer at an advanced stage, risk factors for breast cancer were little observed.

## Introduction

Les cancers du sein constituent le 2^e^ cancer le plus fréquent dans le monde après les cancers broncho-pulmonaires [1]. Il s´agit du 1^er^ cancer chez la femme, touchant 2,1 millions de nouveaux-cas dans le monde en 2018 [1]. Son incidence est plus élevée dans les pays développés alors que le taux de mortalité est plus élevé dans les pays en voie de développement où le cancer du sein est la 1^ère^ cause de décès par cancer [1]. A Madagascar, l´absence d´un registre du cancer rend difficile l´évaluation réelle du poids du cancer du sein dans la population. La majorité des études épidémiologiques sur ces cancers était effectuée dans la capitale, Antananarivo, où le cancer du sein serait le plus fréquent des cancers [2-5]. En effet, les services de prise en charge du cancer à Madagascar se situaient tous dans la capitale jusqu´en 2011; et le deuxième service d´Oncologie du pays a ouvert ses portes à Fianarantsoa. Aussi, à travers notre étude, nous présentons les premiers résultats des aspects épidémiologiques et cliniques sur les cancers du sein à Fianarantsoa. Notre objectif était de décrire les aspects épidémiologiques et cliniques des cancers du sein au Service d´Oncologie du Centre Hospitalier Universitaire de Tambohobe (CHU-T) Fianarantsoa.

## Méthodes

### Conception et cadre de l´étude

Il s´agissait d´une étude rétrospective descriptive, de janvier 2011 à décembre 2018 (08 ans) effectué au Service d´Oncologie du CHU-T. Madagascar possède actuellement 6 Services d´Oncologie dont 2 à Antananarivo, la capitale du pays et les 4 autres dans les 4 autres provinces à savoir: Fianarantsoa, Mahajanga, Toliara et Toamasina. Le service d´Oncologie du CHU-T a ouvert ses portes en décembre 2010. Il s´agit du seul Service d´Oncologie dans la province de Fianarantsoa et est le 2^e^ Service d´Oncologie ouvert à Madagascar. Fianarantsoa est une ville des hautes terres de Madagascar, chef-lieu de la région Haute Matsiatra et se situe à 413 km au Sud d´Antananarivo. Cette région possède deux Centres Hospitaliers Universitaires. La prise en charge thérapeutique du cancer du sein se fait exclusivement auprès du CHU de Tambohobe alors que l´examen cyto-anatomopathologique s´effectue au CHU d´Andrainjato, situé à 4 km du CHU de Tambohobe. Une seule clinique privée de Fianarantsoa possède une mammographie de dépistage.

### Population de l´étude

Nous avons inclus toutes les patientes portant sur les cas de cancers du sein observés ayant une confirmation cytologique et/ou histologique. Les critères d´exclusion étaient les patientes présentant une anomalie mammaire sans confirmation cytologique et/ou histologique de malignité, les tumeurs bénignes du sein et les dossiers incomplets.

### Collecte des données

Les variables étudiées étaient l´âge, la profession, les antécédents familiaux de cancer du sein, l´âge de la ménarche, l´âge de la ménopause, la parité, la notion de prise de traitement contraceptif et le tabagisme. Sur le plan clinique, nous avons collecté: les circonstances de découverte de la maladie, les symptômes mammaires, les signes d´extension loco-régionale et métastatique, la localisation tumorale selon le sein atteint et selon le quadrant atteint, et le stade de la maladie selon la classification de l´*American Joint Committee on Cancer* (AJCC) de 2009. Les données ont été recueillies à partir de la consultation des dossiers médicaux des patientes au sein du Service d´Oncologie. Ces données étaient recueillies sur le logiciel Excel 2003.

### Analyse statistique

Les variables quantitatives étaient exprimées en termes de moyen et les variables qualitatives en termes de pourcentage. La saisie des textes, des tableaux et des graphiques a été traitée sur le logiciel Excel 2003. Les données étaient analysées par le logiciel Epi Info version 7.

### Considération éthique

Nous avons respecté l´anonymat des dossiers traités. Nous avons obtenu l´autorisation du Directeur de l´établissement avant de réaliser l´enquête.

## Résultats

### Caractéristiques sociodémographiques

**Figure 1:**
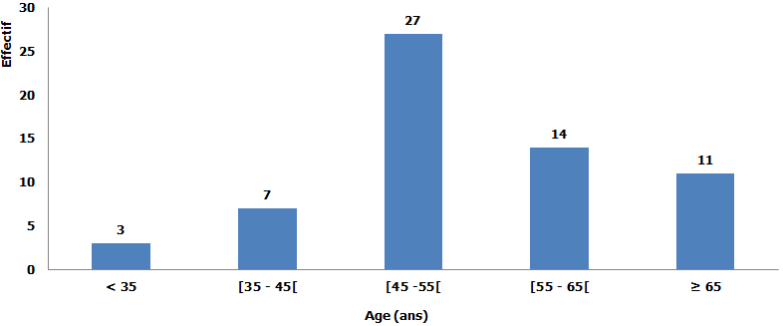
répartition selon l´âge des patientes atteintes du cancer du sein au Service d´Oncologie (2011 à 2018)

**Figure 2:**
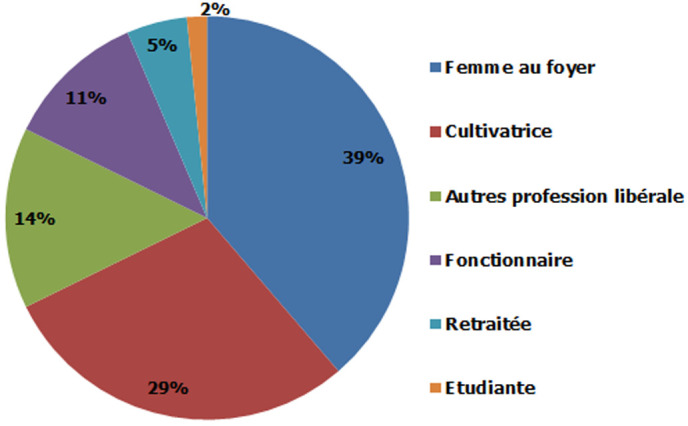
répartition selon la profession des patientes atteintes du cancer du sein au Service d´Oncologie (2011 à 2018)

Durant la période d´étude, 62 patientes ont été incluses. L´âge moyen au diagnostic était de 52,83 +/- 10,47 ans, l´âge minimal était de 28 ans et l´âge maximal de 76 ans (Figure 1). La profession des patientes était constituée par les femmes au foyer dans 39% des cas (n = 24) et les cultivatrices dans 29% des cas (n = 18) (Figure 2).

### Antécédents

L´âge moyen de la ménarche était de 15,14 +/- 1,90 ans et aucune de nos patientes n´avait eu ses premières règles avant l´âge de 11 ans. La nulliparité constituait 6,45% des cas (n = 04) et la parité moyenne était de 3,42 +/- 2,23. Au moment du diagnostic, 39% des femmes (n = 24) étaient ménopausées et 6,45% (n = 04) étaient ménopausées après 55 ans. La contraception orale était utilisée par 25,81% (n = 16) des femmes (Tableau 1). Des antécédents familiaux de cancer du sein ont été retrouvés chez 8,06% des patientes (n = 05). La prise de tabac a été retrouvée chez 14 patientes (22,58%): 3 patientes (4,84%) avaient pris du tabac à fumer et 11 patientes (17,74%) avaient pris du tabac à chiquer.

**Tableau 1:** répartition selon les antécédents des patientes

Variables	Effectif (n = 62)	Pourcentage (%)
**Ménarche**		
≤ 11 ans	0	-
12-15 ans	20	32,26
> 15 ans	11	17,74
Non précisée	31	50,00
Age moyen du ménarche (ans)	15,14 +/- 1,90	
**Parité**		
0	4	6,45
1 à 2	4	6,45
3 à 4	12	19,36
≥ 5	21	33,87
Non précisée	21	33,87
Age moyen de la parité (ans)	3,42 +/- 2,23	
**Statut ménopausique**		
Pré-ménopause	19	30,65
Post-ménopause	24	38,71
Non précisé	19	30,65
**Age à la ménopause**		
≤ 50 ans	12	19,35
50 - 55 ans	8	12,90
> 55 ans	4	6,45
**Contraception orale**		
Oui	16	25,81
Non	28	45,16
Non précisée	18	29,03

### Caractéristiques cliniques

Le mode de découverte de la maladie était la présence de symptômes mammaires dans 97% des cas (n = 60). Les deux autres (3%) modes de découverte de la maladie étaient la présence de signes de localisation secondaire pulmonaire et osseuse. Les symptômes mammaires rencontrés étaient les tuméfactions mammaires dans 95,2% des cas (n = 59) suivies par les douleurs mammaires dans 3,2% (n = 2) et les écoulements mamelonnaires dans 1,6% des cas (n = 1).

Le sein gauche était touché dans 62,90% des cas (n = 39) et le quadrant supéro-externe dans 53,23% des cas (n = 33) (Tableau 2). Selon la classification de l´AJCC de 2009, 55% (n = 34) des cancers du sein ont été diagnostiqués au stade III, 32% (n = 20) au stade IV et 13% (n = 8) au stade I et II. Les signes d´extension loco-régionale de la maladie étaient les adénopathies axillaires homolatérales dans 51,61% des cas (n = 32) et les signes métastatiques étaient peluro-pulmonaires dans 16,13% des cas (n = 10) (Tableau 3).

**Tableau 2:** répartition selon la localisation tumorale

Variables	Effectif (n = 62)	Pourcentage (%)
**Sein envahi**		
Sein droit	23	37,10
Sein gauche	39	62,90
**Quadrant envahi**		
Quadrant supéro-externe	33	53,23
Quadrant supéro-interne	7	11,29
Quadrant inféro-externe	5	8,06
Quadrant inféro-interne	4	6,45
Biquadrant	4	6,45
Tout le sein	9	14,52
Région péri-mamelonnaire	0	-

**Tableau 3:** répartition selon les signes d´extension loco-régionale et à distance

Signes d´extension	Effectif (sur 62 cas)	Pourcentage (%)
**Extension loco-régionale**		
Ganglion axillaire homolatérale	32	51,61
Peau d´orange	7	11,29
Rétraction mamelonnaire	6	9,68
Nodules de perméation	4	6,45
Ganglion sus-claviculaire	2	3,23
Ganglion axillaire controlatérale	0	0,00
**Métastases à distance**		
Image en lâcher de ballon	9	14,52
Masse pulmonaire	2	3,23
Epanchement pleural	1	1,61
Tassement vertébral	1	1,61
Lyse osseuse	1	1,61
Masse cérébrale	1	1,61
Compression médullaire	1	1,61
Nodules hépatiques	1	1,61
Ascite	0	0,00

## Discussion

En huit ans, nous avons colligé 62 cas soit une fréquence annuelle de 7,75 cas par an. A Antananarivo, durant l´année 1996 à 1998, 259 cas ont été inclus [2] et durant l´année 2007 à 2010, 189 cas ont été inclus [6]. A l´hôpital militaire de Soavinandriana, 75 cas ont été inclus en 25 mois [7]. Dans les pays africains, 330 cas ont été inclus en 5 ans au Ghana et 212 cas en 08 ans au Nigéria [8, 9]. Le nombre de cas de patientes atteintes de cancer du sein à Fianarantsoa serait moins élevé par rapport aux autres études malgaches et africaines probablement lié à l´absence de confirmation cyto-anatomopathologique de la majorité des cas suspectés.

Dans notre étude, l´âge moyen au diagnostic était de 52,83 ans. A Antananarivo entre 1995 et 2014, l´âge moyen au diagnostic variait de 48,5 ans à 50,73 ans [2-5, 7]. Au Ghana et au Nigéria, l´âge moyen était de 49,19 et de 48 ans [8, 9]. Le faible effectif de notre échantillon pourrait expliquer cette différence d´âge. Dans les pays développés, l´âge moyen au diagnostic était plus élevé variant de 62,3 à 63,3 ans [10]. La raison la plus probable est la faible espérance de vie de la population africaine. Selon l´Organisation Mondiale de la Santé, l´espérance de vie de la population malgache est de 66,1 ans contre 78,5 ans aux Etats-Unis et 82,9 ans en France [11].

Dans notre étude, les femmes au foyer prédominaient (39%) suivies par les cultivatrices (29%). La prédominance des femmes au foyer (56,5%) était aussi retrouvée par Balekouzou *et al*. [12]. Les fonctionnaires étaient minoritaires dans notre étude car elles préfèrent probablement aller dans la capitale où le coût des bilans sont pris en charge par l´Etat alors qu´à Fianarantsoa, elles seraient obligées de payer ces bilans. Une facilitation de l´accès aux soins pour ces personnes devrait augmenter notre recrutement. Les facteurs de risque connus du cancer du sein [13] étaient peu observés chez nos patientes. Seules 3,84% des patientes avaient une ménarche précoce. Au Burkina Faso, seules 2,50% des patientes avaient eu leur ménarche avant 11 ans [14] et selon Nzeangung *et al*. seules 3,1% des femmes avaient une ménarche précoce [15]. Dans notre étude, 3,84% des femmes étaient nullipares. Dans des études africaines, la nulliparité ne s´observait que chez 4,2 à 6,25% des patientes [9, 14]. Des études concernant la relation entre ces facteurs et la survenue de cancer du sein chez les femmes malgaches devraient être menées.

Dans notre étude, 30,65% des patientes n´étaient pas ménopausées au moment du diagnostic. Dans d´autres études africaines, un taux plus élevé de personnes non ménopausées était retrouvé chez 46,25 à 67% des patientes [2, 9, 14, 15]. Les femmes en préménopause seraient plus touchées dans la population africaine alors que dans notre étude, les femmes ménopausées étaient plus fréquentes. Le statut ménopausique imprécis chez 30,65% de nos patientes limite notre étude.

La contraception hormonale était utilisée par 25% de nos patientes. Un taux plus élevé d´utilisation de la contraception orale était rapporté au Nigéria et au Burkina Faso avec 28,6% et 36,25% respectivement [9, 14]. Même avec l´utilisation des nouveaux contraceptifs oraux microdosés, il existe un risque modéré de survenue du cancer du sein [16, 17]. Les modifications des habitudes de vie permettraient de diminuer le risque de cancer du sein et de contrebalancer les effets de la contraception hormonale orale [16]. Le planning familial faisant partie intégrante de la politique nationale de santé à Madagascar [18], d´autres études devront être menées pour connaître leur rôle dans la survenue du cancer du sein chez les femmes Malgaches.

Cinq patientes (9,62%) avaient un antécédent familial de cancer du sein. Au Nigéria, 7,2% des patientes avaient un antécédent familial de cancer mammaire [9]. Toute femme avec un antécédent familial de cancer du sein devrait être se faire dépister régulièrement. Onze patientes (17,31%) consommaient du tabac à chiquer. Selon l´*International Agency for Research on Cancer*, le tabac non fumé est cancérigène pour l´homme [19]. Selon Spangler *et al*. l´utilisation des tabacs non fumés aurait un lien avec le cancer du sein aux Etats-Unis [20]. Une exploration d´un lien entre le tabac à chiquer et le cancer du sein chez la population Fianaroise serait intéressante pour orienter les mesures de prévention. Les symptômes mammaires rencontrés dans notre étude étaient dominés par les tuméfactions mammaires (95,2%) suivies par les douleurs mammaires (3,2%) et les écoulements mamelonnaires (1,6%). Au Ghana, les tuméfactions mammaires prédominaient aussi (75,2%) [8]. Les tuméfactions mammaires seraient le principal symptôme des cancers du sein d´où la nécessité de sensibiliser les femmes à consulter dès la présence de ce symptôme.

Nos cancers du sein étaient diagnostiqués à un stade avancé (87%). Ce taux est élevé comparé aux autres pays en développement où les stades avancés constituent 30 à 80% des cas [6-8, 10, 21]. Dans les pays développés, les stades avancés sont moins importants [10, 21]. En Australie et en Angleterre, 55,9% et 56,8% des cancers du sein sont découverts à un stade localisé [10]. L´insuffisance de dépistage à Fianarantsoa et le retard de consultation pourraient contribuer à la survenue de ces stades avancés. Nous ré étirons ici l´importance d´une sensibilisation des femmes à venir consulter précocement en présence de symptômes mammaires.

Parmi les signes d´extension loco-régionale, nos résultats rejoignent ceux d´Adesunkanmi *et al*. [9], retrouvant une prédominance des adénopathies axillaires (51,61%). Les métastases pulmonaires étaient la localisation métastatique la plus fréquente (19,36%) dans notre étude rejoignant les résultats d´Adesunkanmi *et al*. (20,3%) [9] et pour Nzeangung *et al*. la localisation pulmonaire était la deuxième localisation métastatique après les métastases osseuses [15]. Devant tout cancer du sein, l´exploration pulmonaire devrait être systématique.

Ce cancer prédominait au niveau du quadrant supéro-externe (53,23%) concordant avec les données de la littérature. Le quadrant supéro-externe était le principal site de prédilection des cancers du sein dans 40 à 69,5% des cas [9, 22, 23] et serait lié à l´importance du tissu mammaire à ce niveau [23, 24]. La présence de signes mammaires au niveau de ce quadrant devrait faire penser en premier lieu à un cancer du sein.

## Conclusion

Le cancer du sein de notre centre touche une population plus âgée que dans les études malgaches antérieures. Les principaux facteurs de risque établis étaient peu retrouvés. Le principal symptôme mammaire était les tuméfactions mammaires. La majorité des cas était diagnostiqué à un stade avancé. Faciliter l´accès au dépistage et sensibiliser les femmes à une consultation précoce dès la présence de tuméfactions mammaires permettraient de réduire le nombre de stade avancé de la maladie. Une exploration plus approfondie d´autres facteurs de risque serait opportune afin d´établir une action de prévention primaire de ces cancers.

### Etat des connaissances sur le sujet

- Le cancer du sein touche les sujets jeunes à Madagascar;
- Ces cancers sont diagnostiqués à un stade avancé de la maladie en l´absence de dépistage.

### Contribution de notre étude à la connaissance

- Les femmes atteintes du cancer du sein étaient plus âgées que dans les études malgaches;
- Les facteurs de risque connus de ces cancers étaient peu observés;
- Malgré la présence d´un dépistage à Fianarantsoa, les cancers du sein restent de diagnostic tardif.
